# Establishment of mitochondrial pyruvate carrier 1 (MPC1) gene knockout mice with preliminary gene function analyses

**DOI:** 10.18632/oncotarget.13210

**Published:** 2016-11-08

**Authors:** Xiaoli Li, Yaqing Li, Gaoyang Han, Xiaoran Li, Yasai Ji, Zhirui Fan, Yali Zhong, Jing Cao, Jing Zhao, Goscinski Mariusz, Mingzhi Zhang, Jianguo Wen, Jahn M. Nesland, Zhenhe Suo

**Affiliations:** ^1^ Department of Oncology, The First Affiliated Hospital of Zhengzhou University, Zhengzhou, Henan Province, China; ^2^ Department of Pathology, The Norwegian Radium Hospital, Oslo University Hospital, Institute of Clinical Medicine, University of Oslo, Montebello, Oslo, Norway; ^3^ Department of Thoracic Surgery, The First Affiliated Hospital of Xinxiang Medical University, Weihui City, Henan Province, China; ^4^ Department of Pathology, The Third Affiliated Hospital of Zhengzhou University, Zhengzhou, Henan Province, China; ^5^ Department of Surgery, The Norwegian Radium Hospital, Oslo University Hospital, Institute for Clinical Medicine, Faculty of Medicine, University of Oslo, Norway; ^6^ The Institute of Clinical Medicine, The First Affiliated Hospital of Zhengzhou University, Zhengzhou, Henan Province, China

**Keywords:** MPC1, CRISPR/Cas9, diabetes, gene knockout, reproductive capability

## Abstract

Pyruvate plays a critical role in the mitochondrial tricarboxylic acid (TCA) cycle, and it is the center product for the synthesis of amino acids, carbohydrates and fatty acids. Pyruvate transported across the inner mitochondrial membrane appears to be essential in anabolic and catabolic intermediary metabolism. The mitochondrial pyruvate carrier (MPC) mounted in the inner membrane of mitochondria serves as the channel to facilitate pyruvate permeating. In mammals, the MPC is formed by two paralogous subunits, MPC1 and MPC2. It is known that complete ablation of MPC2 in mice causes death on the 11th or 12th day of the embryonic period. However, MPC1 deletion and the knowledge of gene function *in vivo* are lacking. Using the new technology of gene manipulation known as Clustered Regularly Interspaced Short Palindromic Repeats/CRISPR-associated 9 (CRISPR/Cas9) systems, we gained stable MPC1 gene heterozygous mutation mice models, and the heterozygous mutations could be stably maintained in their offsprings. Only one line with homozygous 27 bases deletion in the first exon was established, but no offsprings could be obtained after four months of mating experiments, indicating infertility of the mice with such homozygous deletion. The other line of MPC1 knockout (KO) mice was only heterozygous, which mutated in the first exon with a terminator shortly afterwards. These two lines of MPC1 KO mice showed lower fertility and significantly higher bodyweight in the females. We concluded that heterozygous MPC1 KO weakens fertility and influences the metabolism of glucose and fatty acid and bodyweight in mice.

## INTRODUCTION

Pyruvate is a critical product in the metabolism of glucose and plays a central role in the synthesis of fatty acids and non-essential amino acids [1]. Physiologically, pyruvate may be converted to lactate acid through glycogenesis, or be converted into acetyl-coenzyme A under the action of pyruvate dehydrogenase, entering the tricarboxylic acid cycle for mitochondrial ATP generation [2]. Theoretically, mutations in any of the genes participating in the pyruvate metabolism may result in abnormal glycolysis and tricarboxylic acid cycle of cells. Although there are many animal model studies focusing on the metabolism-related enzymes, germ-line mutation of mitochondrial pyruvate carrier (MPC) animal model is still lacking.

It is known that pyruvate, as well as some molecules, can freely diffuse the outer-membrane of mitochondria, but requires a specific carrier to transit to the inner mitochondrial membrane and reach the mitochondrial matrix [3]. It was demonstrated the existence of carrier-assisted transportor of pyruvate across the inner mitochondrial membrane by Papa and Halestrap in 1970's [4, 5]. However, the protein responsible for the mitochondria pyruvate transport was later identified in yeast in 2003, and further characterized in mammalian cells in 2012 [6–8]. In mammals, MPC complex is a hetero-oligomeric complex consisting of MPC1 and MPC2 which mounting in the inner mitochondrial membrane and it facilitates pyruvate into the mitochondrial matrix. Studies show that MPC1 mutations cause illness, including lactic acidosis, hyperpyruvatemia and other severe diseases, which lead to short life span in humans [6, 7]. Such roles of pyruvate metabolism were confirmed in other studies [9–11].

Studies have shown that MPC activity is positively associated with glucagon or fatty acid and negatively associated with insulin levels [12–14]. The disorder of pyruvate accumulation may influence the metabolism and thus result in the disorder of the lipid metabolism, including obesity. Obesity and its associated disorders are the increasing health challenges, especially in the Western world [15]. Therefore, molecular biological mechanism studies have contributed to the development of molecular therapeutic strategies against obesity and diabetes [16]. It is clear now that the mitochondrial pyruvate metabolism performed by pyruvate dehydrogenase and pyruvate decarboxylase plays an important role in regulating the insulin release [17], which may be the potential cause of the high rate morbidity of diabetes.

In addition, it has been shown that the ablation of MPC2 in mice causes death on the 11th or 12th day of the embryonic period [18], indicating the important role of MPC2 in the embryonic development. Because there is still limited knowledge of MPC function, loss of MPC gene function experiments *in vivo* may help to better understand the role of MPC and to disclose drug discovery possibility for metabolic diseases. Various skills can be used to knock out or knock in any gene, thus forming truncated or extended gene sequences, among which several efficient gene-modifying tools thrive all around the world. Clustered Regularly Interspaced Short Palindromic Repeats (CRISPR) systems derived from bacteria have enabled the development of targeted genome editing. CRISPR RNAs (crRNAs) and CRISPR-associated (Cas) proteins, which were two important parts of this system, enable the CRISPR systems to direct the degradation of complementary sequences [19–21]. Reconstitution of the streptococcus pyogenes type II CRISPR system *in vitro* has shown that crRNA correctly fuses with the target DNA sequence, and that the Cas9 protein plays a role in cleaving the matching DNA [22]. Experiments in the eukaryotic organisms of yeast, plants and even mammals have demonstrated that this complex system plays a great role in gene editing [22, 23].

The purposes of our current study were to explore whether MPC1 gene knockout (KO) mice could be established with the CRISPR technology and to obtain general insight of the gene mutation in mice.

## RESULTS

### Variable MPC expressions are present in different tissues in the C57BL/6J mice

**Figure 1:**
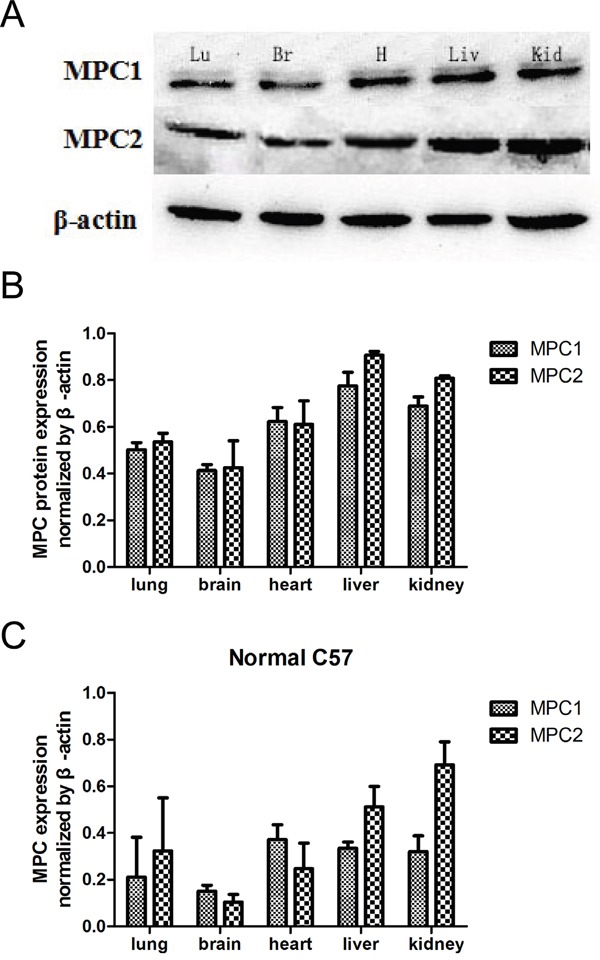
MPC1 and MPC2 expressions in multiple organs and Western blotting results **A.** Western blotting shows variable expressions of MPC1 and MPC2 in different organs. **B.** Corresponding quantified densitometry of the Western blotting results as shown in (A). Date are presented as mean ± SEM (n=3 separate animals). **C.** Relative MPC mRNA levels in the indicated mouse tissues. Values are presented as mean ± SEM (n=3 separate animals). Br, brain; Lu, lung; H, heart; Liv, liver; Kid, kidney.

Western blotting analysis of tissue lyses demonstrated that variable MPC1 and MPC2 protein expressions were present in multiple tissues of the wild type C57BL/6J mice (Figure 1A, 1B). Similar mRNA expressions of MPC1 and MPC2 were also verified in all tissues with quantitative RT-PCR (Figure 1C), suggesting a fundamental role of MPC in mice. In general, higher levels of MPC1 expression were discovered in the liver, kidneys and heart, with approximately 55% higher expression than that in the lung and brain. MPC2 was highly expressed in the liver and the kidneys as well. Considering all of these findings, we chose mouse liver tissues for the subsequent experiments in order to follow the expression alterations in the MPC1 mutation mice.

### MPC1 gene KO mice verification

**Figure 2:**
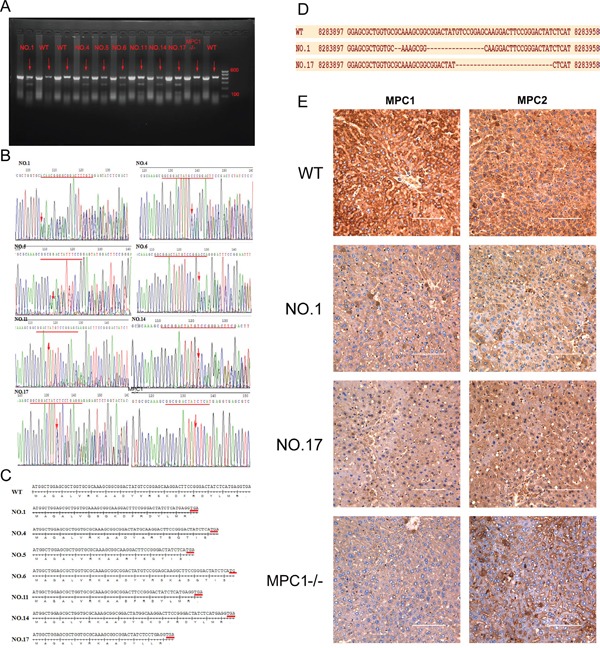
T7E1 assay, sequencing and Western blotting used for identification of gene KO mice **A.** The mutagenesis of the target sites is revealed by the presence of a full-length PCR product with digestion products of 278 and 150 bps (Marker shows in the picture from top to bottom: 600bp, 500bp, 400bp, 300bp, 200bp, 100bp). **B.** Sequence data of mutations in several mutation mice. Red lines indicate deletion of target(s). The red arrows indicate the beginning sites of deletion. **C.** The deleted base sequences of each heterozygous model relative to the WT one. **D.** shows single strand DNA sequences of WT, NO.1 and NO17 heterozygous mutation mice. **E.** shows results of IHC, on which the WT mice show strong expression of MPC1 and MPC2 proteins, while the NO1 and NO17 mice show reduced levels of the expression of MPC1 and MPC2 proteins, and the MPC1−/− mouse shows very weak MPC1 protein expression, but relatively weak MPC2 protein expression. Values are presented as mean ± SEM (n=3 separate animals).

Two F0 foster mice harboured MPC1-g1 and MPC1-g2 plasmids on their embryos. Of the 17 descendants, 12 displayed a heterozygous mutation, five from the MPC1-g1 plasmid and seven from the MPC1-g2 plasmid (Table 1). Successful knockout of the MPC1 gene was identified by the T7 endonuclease 1 (T7E1) mismatch sensitive assay, based on the principle that if the duplexes formed contained a mutation, it could be digested by the T7E1 enzyme as shown by the two small fragments in Figure 2A. Further sequencing of the products revealed seven different mutations, which was identified in the mice of Nos. 1, 4, 5, 6, 11, 14 and 17, respectively (Figure 2B).

**Table 1:** Genotype distribution at weaning

pseudopregnant foster mice	Target sites	Total Pups	WT	Heterozygotes KO
1	MPC1-g1	7	2	5
2	MPC1-g2	10	3	7

The sequences of each mutation and the deduced peptide sequences are shown in Figure 2C and 2D. Compared to the wild type (WT) mice, all the mutations were located in the first exon and created an early terminator, which resulted in a very short transcript as shown in Figure 2C. In line with the above findings, immunohistochemistry (IHC) of the MPC1 and MPC2 in the liver of the MPC1 mutation mice also revealed protein expression differences. As shown in Figure 2E, decreased MPC1 protein expression is seen in the livers of the No.1 and No.17 MPC1 heterozygous KO mice, while the MPC1 protein expression in the MPC1 homozygous KO (MPC1−/−) mice was further decreased. Intriguingly, the MPC2 protein expression in the MPC1 heterozygous and homozygous mice was also correspondingly decreased.

**Figure 3:**
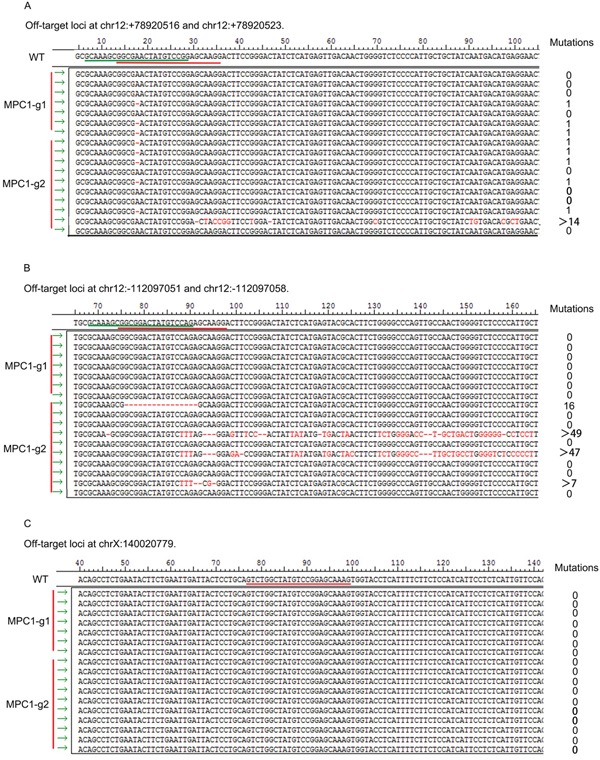
Off-target analyses **A.** Mutations at loci chr12:+78920516 and chr12:+78920523. Three of the seven F0 MPC1-g1 mice showed a single base deletion, and 6 of the 10 MPC1-g2 mice revealed variable mutations, 5 mice with a single base deletion and another with multiple mutations. **B.** Mutations at the loci at chr12:-112097058 and chr12:-112097051. There was no any mutation discovered for the MPC1-g1. However, 4 of the 10 MPC1-g2 mice showed variable mutations. **C.** Mutations at the locus at chrX:+140020779. There was no any mutation for both the MPC1-g1 and MPC1-g2.

For the off-target verification, we performed PCR of the F0 mice tail DNA with the specific primers covering the MPC1-g1 and MPC1-g2 off-targets. For the off-targets at locus chr12:+78920516 and chr12:+78920523, it was found that 3 of the 7 F0 MPC1-g1 mice showed a single base deletion, and 6 of the 10 MPC1-g2 mice revealed variable mutations, 5 mice with a single base deletion and another with multiple mutations as shown in Figure 3A. For the off-targets at locus chr12:-112097058 and chr12:-112097051, there was no any mutation was discovered for the MPC1-g1. However, 4 of 10 MPC1-g2 mice showed variable mutations as shown in Figure 3B. For the off-target at chrX:+140020779, no any mutation for both the MPC1-g1 and MPC1-g2 was revealed as shown in Figure 3C.

### Changes of metabolism genes

**Figure 4:**
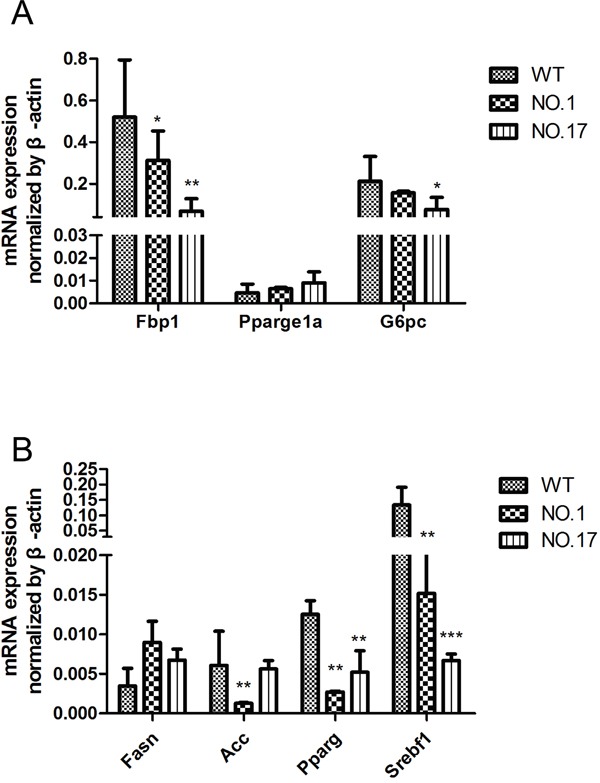
Metabolism genes influence by the heterozygous MPC1 gene KO in the liver **A.** qRT-PCR detection of gluconeogenesis genes expression in WT and the heterozygous MPC1 gene KO mice. **B.** Relative lipogenesis genes expression in WT and the heterozygous MPC1 gene KO mice. Values are presented as mean ± SEM (n=3 separate animals, ^*^<0.05, ^**^<0.01, ^***^<0.001).

As pyruvate plays a central role in glucose and lipid metabolism, we speculated that the MPC1 dysfunction may alter other metabolism-related gene expressions. We therefore measured the expression of metabolism-related genes, which are partly linked to the function of the *MPC1* gene, including those involved in gluconeogenesis (*Fbp1, G6pc, Ppargc1a*) and lipogenesis (*Acc, Pparg, Fasn, Srebf1*). Some of the enzymes for gluconeogenesis and lipogenesis decreased significantly, while others had no change (Figure 4A and 4B).

### Fertility impact of MPC1 KO

In order to gain a stable mutation line of mice, different F0 mice were mated with corresponding WT counterparts, and the mating experiments were repeated for six generations. After long-term mating experiments, only two lines of mice derived from No.1 and 17 mice were maintained. The No.1 mice were developed from the MPC1-g1 plasmid transfection, while the No.17 mice were developed from the MPC1-g2 plasmid transfection. With the descendants of these two lines, further mating experiments were performed and the results are shown in Table 2. Of note, five No. 17 descendants were identified as MPC1 homozygous KO (MPC1−/−). With these five MPC1−/− mice, mating experiments between MPC1−/− and MPC1−/− mice, and between MPC1−/− and WT mice were conducted for four months, but no single pregnancy was observed, highlighting the infertility of the homozygous MPC1 gene in KO mice. These experiments also revealed a decreased fertility in the heterozygous MPC1 KO mice.

**Table 2:** The fertility of MPC1 KO mice

Gene Type	Male	Female	Mating NOS.	Pregnancy percentage	Number of Offsprings		
					Total	Hetero-zygote	Homo-zygote
WT	WT	WT	15	100%(14/14)	102		
NO.1	WT	MPC1+/−	12	75%(9/12)	26	8(31%)	0
	MPC1+/−	WT	13	46%(6/13)	23	6(26%)	0
	MPC1+/−	MPC1+/−	13	46%(6/13)	34	12(35%)	0
NO.17	WT	MPC1+/−	15	60%(9/15)	76	38(50%)	0
	MPC1+/−	WT	13	62%(8/13)	64	30(47%)	0
	MPC1+/−	MPC1+/−	15	73%(11/15)	73	27(37%)	5(7%)
	WT	MPC1−/−	5	0%(0/5)	0	0	0
	MPC1−/−	WT	4	0%(0/4)	0	0	0
	MPC1−/−	MPC1−/−	4	0%(0/4)	0	0	0

### Bodyweight influence of MPC1 KO

**Figure 5:**
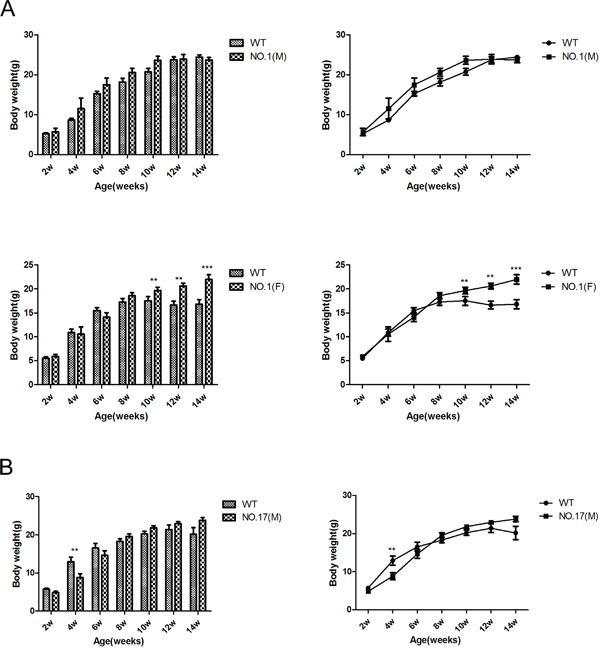
Effect of different heterozygous MPC1 KO on bodyweight **A.** Quantitative display of the bodyweight in NO.1 compared with WT mice. Upper part shows the bodyweight influence on the male mice, and the lower part shows the bodyweight influence on the female mice. **B.** Quantitative display of the bodyweight gain in NO.17 compared with WT mice. Upper part shows the bodyweight influence on the male mice, and the lower part shows the bodyweight influence on the female mice. Values are presented as mean ± SEM (n=7 separate animals, ^**^<0.01, ^***^<0.001).

The mitochondrial localization of MPC1, which support the normal metabolism, led us to hypothesize that the bodyweight of the MPC1 KO mice may be influenced by the altered MPC function. Metabolism-related phenotypes have been shown to be sex-related, probably due to the different hormone profiles of males and females [25, 26]. There was no bodyweight difference between the No.1 and No.17 derived MPC1 KO male mice, compared to the control mice. However, a significantly higher bodyweight in the No.1 and No.17 derived MPC1 KO female mice was discovered (Figure 5).

### Glucose and insulin tolerance tests of MPC1 heterozygous KO

MPC1 is a carrier for pyruvate, which promotes the main metabolism of glucose. MPC1 KO mice may thus manifest glucose metabolism problems. We hypothesized that the MPC1 KO mice were able to utilize more glucose for energy production than the WT mice, since the increased glycolysis in the mitochondrial function depressed tumour cells usually leads to reduced ATP production as known as Warburg effect [27].

**Figure 6:**
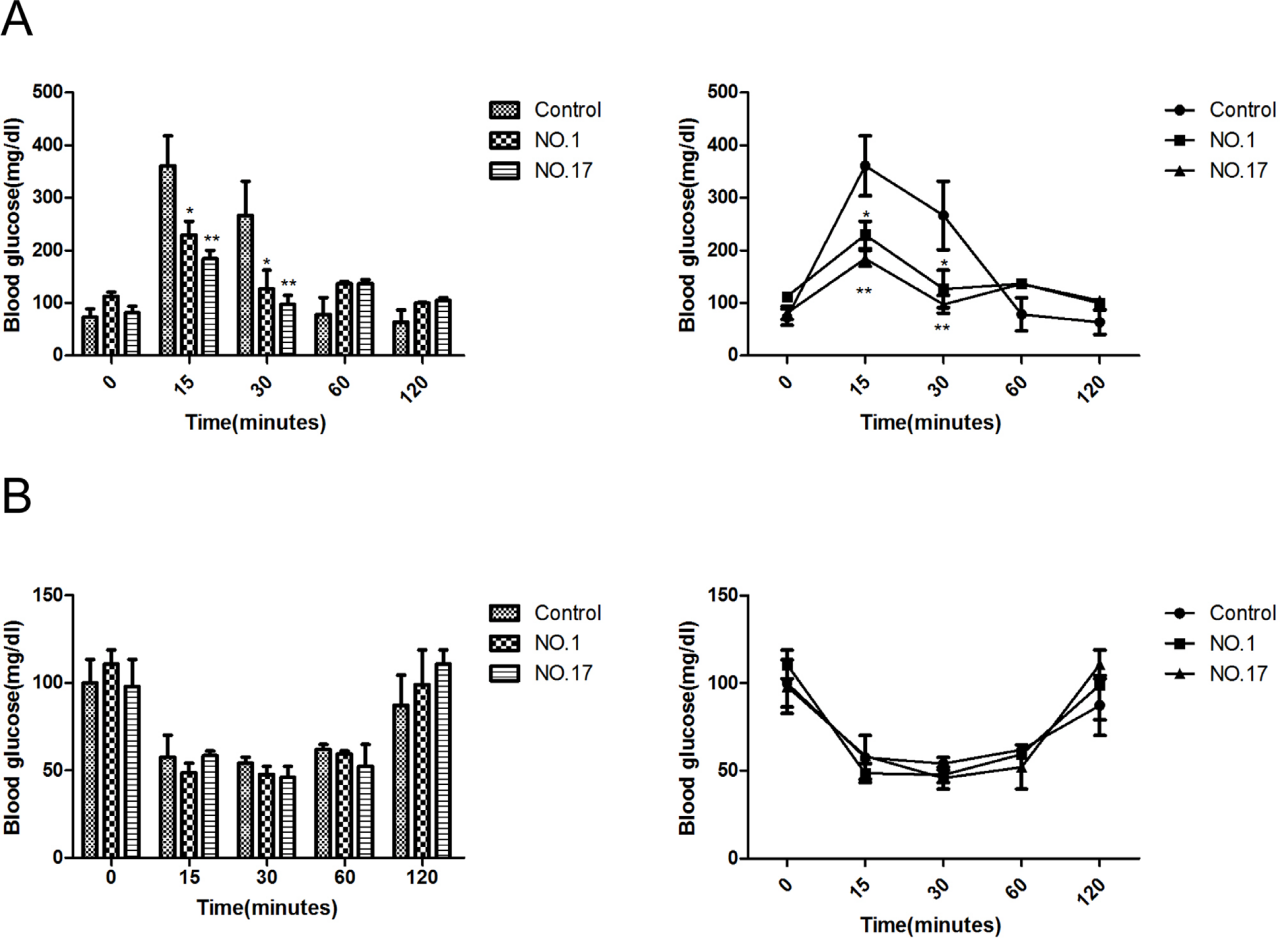
MPC1 KO mice show similar use and storage of carbohydrates **A.** Glucose tolerance test (GTT) in MPC1 KO and WT mice fed in normal food (n = 3). **B.** Insulin tolerance test (ITT) in MPC1 KO and WT mice fed in normal food (n = 3, ^*^<0.05, ^**^<0.01).

As shown in Figure 6A, there is a trend of enhanced glucose tolerance after 15 minutes and 30 minutes treatments in the MPC1 KO female mice, in contrast with the reduced glucose sensitivity typically observed in the obese patients as reported before [28]. Furthermore, upon injection of insulin, the MPC1 heterozygous KO mice showed the same glucose levels as WT mice (Figure 6B). There was no significant difference in the insulin tolerance between the male MPC1 heterozygous KO and WT mice. However, we noted that after overnight fasting, the MPC1 heterologous KO mice fed with standard food had higher glucose levels compared with the WT mice, indicating decreased glucose utilization in the MPC1 heterozygous KO mice during fasting compared to the WT mice.

## DISCUSSION

Studies have shown that CRISPR/Cas9 system was an efficient tool for genome engineering in zebra fish, rats, mice and others [29–32]. The 20 nucleotides spacer sequence and protospacer adjacent motif (PAM) composed by the three nucleotides NGG (N being A, C, G or T) determine the specificity of Cas9 targeting [33]. This gene engineering causes double-strand breaks (DSBs) by recognizing targeting sequences. Repairing DSBs by using the non-homologous end-joining (NHEJ) mechanism may recover small deletions. Moreover, homologous recombination (HR) repairs DSBs with the presence of a donor template [34–35]. The CRISPR/Cas9 system has rapidly promoted the use and speed of gene manipulation for multiple species. After a long trial period, we successfully bred MPC1 heterozygous KO mice by using the CRISPR/Cas9 technology. Although CRISPR/Cas9 system is a highly efficient tool for performing genome editing, off-target mutation is still one major concern. Some mutations in some off-target sites were identified in the 17 F0 mice. However, we only succeeded in maintaining two lines of heterozygous MPC1 KO mice, namely the mice No.1 and No.17. From the PCR product sequencing of F0 mice tail DNA, neither No.1 nor No.17 mice showed any mutation. Therefore, we concluded that there was no off-target mutation in the two mice lines, which were further examined in this study. This is in line with the finding that it is rare to see off-targets in animals [36]. We did not manage to develop any line in all the F0 mice with off-target mutation. However, it is currently unknown for us whether there is any off-target influence on the heterozygous KO line establishment.

The transportation of pyruvate from cytoplasm to mitochondria through the mitochondrial inner membrane has been a hot topic since 2012, after the two groups identified the two carriers for pyruvate transportation in mammals [7, 8]. Both teams characterized the inner membrane proteins, which were initially known as BRP44L and BRP44. In humans, the full length of MPC1 genomic DNA is 18095bp, which contains five exons and locates on 6q27, while MPC2 has 20312bp, comprises five exons and locates on 1q24.2. MPC1 and MPC2 have two transmembrane α helixes in the mitochondrial membrane, which together constitute a heterodimer. The complex proteins have a relative molecular mass of approximately 150kDa. Although the function of the MPC1 and MPC2 is largely characterised [3, 7, 8], the importance of MPC1 gene mutations *in vivo* is still a matter of studies. In our study, there was little phenotype difference between the MPC1 heterozygous KO mice and the WT mice, such as the fur colour or exercise endurance. These mice grew healthy without major developmental flaws, which may reflect the real MPC1 alteration status in some “healthy” humans. However, MPC1 heterozygous mutation causes glucose and fatty metabolism gene changes as we have shown in this study. The abundance of transcripts for de novo lipogenesis was decreased compared to WT mice. This finding is contradictory to the bodyweight gain in the female heterozygous MPC1 KO mice, suggesting that increased de novo lipogenesis enzyme activities may also play roles in these mice in order to balance the gene activity decrease, a hypothesis meriting further studies.

Compared to the pyruvate dehydrogenase (PDH) complex mutation in patients, a similar phenotype caused by MPC mutation has been reported in patient [6]. The lack of PDH epidemiology makes it difficult to diagnose potential idiopathic pyruvate metabolism defects caused by MPC mutation [37]. According to the records, three families have suffered from the pyruvate transport deficiency disease due to the mutations in MPC1. It was reported that the first patient had a homozygous R97W mutation in MPC1, and DNA sequencing revealed a c.289C→T. The patient manifested a severe developmental delay and died at an age of 19 months [6]. The second patient had a L79H change in MPC1 and DNA sequencing confirmed the mutation as c.236T→A [7]. Consistent with the above reports, our study shows that the homozygous MPC1 mutation mice can be born, but they all have certain pathology characteristics, which resulted in a short life span and infertility.

Since pyruvate is a critical metabolism product required in metabolic pathways, it was not unexpected that the ablation of MPC1 resulted in embryonic lethality. Mating with various MPC1 heterozygous KO mice resulted in only five mice displaying a homozygote mutation, and the overall birth rate was much lower than the one observed in the WT mice. In addition, all these homozygous KO mice were dead within 6 months. MPC1 heterozygous mutation mice were phenotypically indistinguishable from the WT litter mates in some of the parameters we examined. But we realize that MPC may regulate the insulin secretion in response to both glucose accumulation and fatty acids catabolism, since its function may determine the fate of pyruvate either into cytoplasmic glycolysis or into mitochondrial oxidative phosphorylation. The accumulation of fatty acids in the body results in weight changes in phenotype. As shown in our research, the MPC1 heterozygous KO female mice were heavier than the wild type at 10 weeks of age. But the MPC1 heterozygous KO male mice weighed the same as the WT male mice, thus reflecting a sex impact mutation difference, for which there is no clear explanation yet.

It has been reported that diabetes drugs thiazolidinediones increase insulin sensitivity by special targeting to a site (mTOT), which in turn inhibits MPC [9]. In the current study, we tried to indirectly explore the relationship between the pyruvate transportation and insulin secretion regulation. After 15 minutes and 30 minutes glucose injection, the MPC1 heterozygous KO mice showed a trend of lower glucose level, which means improved glucose tolerance, relative to the WT mice. But after 30 minutes, the integration trend was increased, which is consistent with the weakened consumption of glucose in the MPC2 knockout mice model reported by McCommis [14]. In the intraperitoneal insulin tolerance test (IPITT) study, the MPC1 heterozygous KO mice responded with the same decrease in glucose levels as WT mice. However, the MPC1 heterozygous KO mice had higher glucose levels after overnight fasting compared to the WT mice while completing the IPGTT or IPITT preparation, indicating decreased glucose utilization by the MPC1 heterozygous KO mice during fasting as compared to WT mice. It would be worth to evaluate the effect of the heterozygous MPC1 KO in pancreas, to see how insulin secretion and blood glucose consumption are influenced by the heterozygous MPC1 KO in future studies. However, our current results are largely consistent with the findings in conditional liver MPC1 knockout mice [38].

In the current study, we constructed a pyruvate transport deletion mice model by knocking out the MPC1 gene, which will be useful for further study of the MPC function *in vivo*. The MPC1 heterozygous mutation mice mating experiments have confirmed the reduced fertility capacity, which is consistent with the lethal MPC2 deletion in embryos. It is reasonable to speculate that MPC1 and MPC2 play the same role in the pyruvate cellular transportation in mice.

In conclusion, this study provides the description of MPC1 KO mice generation, and heterozygous MPC1 KO weakens fertility and influences the metabolism of glucose and fatty acid and bodyweight in mice.

## MATERIALS AND METHODS

### Ethics statement

C57BL/6J mice were housed in a temperature controlled room at about 25°C in an animal facility and bred under specific pathogen-free conditions with autoclaved (sterile) food and sterile filtered water, the light-dark cycle at the animal centre laboratory of Henan province is a 12:12 hour. The animal study was admitted by the Institutional Animal Care and Use Committees of the China Medical Association (Permit Number: SCXK 2013–0001).

### Construction of CRISPR/ Cas 9 plasmids and activity analysis

**Figure 7:**
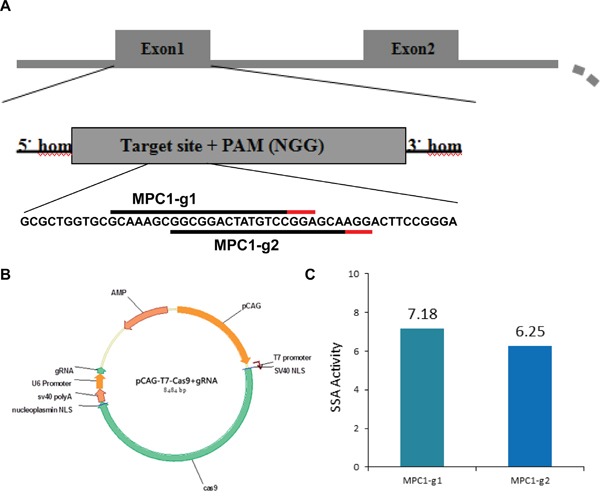
Genome editing in mouse embryo using CRISPR/Cas9 system **A.** Mouse MPC1 loci and the locations of the two protospacer within the first exon of the MPC1 genes. The 20bp protospacers on the sense strand for the MPC1-g1 and MPC1-g2 are indicated by black lines and the adjacent PAM sequences are indicated by the red lines. **B.** RNA-guided gene targeting in mouse embryo includes the Cas9 and gRNA, which are two important parts and formed an integrated circle with correspondence promoters. The gRNA is specific recognizing the target and the Cas9 cleave the DNA chain. **C.** Histogram shows high efficiencies of CRISPR-mediated nuclease activity at the target loci.

The Cas9/sgRNA expression vector was purchased from Nuolanxin Bio-Chemistry Co. Ltd., Beijing, China. Mouse MPC1 gene sequence was retrieved from the gene bank. Two MPC1 exon 1 targets were selected and named as MPC1-g1 and MPC1-g2 (Figure 7A). Two plasmids were constructed as one circle plasmid, and the structure of the constructed plasmids is shown in Figure 7B. Then, by using the circle plasmid, sgRNAs and Cas9 mRNA were transcribed by sgRNA and Cas9 expression vectors and the MEGAshortscript™ Kit (Ambion, AM1345). Following transcription completion, poly (A) tailing reaction and DNase I (TURBO, AM2238) treatment were performed. The sgRNA and the Cas9-encoding mRNA were then purified by MEGAclear™ Kit (Ambion, AM1908) and re-dissolved in RNase-free water. The luciferase SSA analyses were carried out to confirm the plasmids' activity, which showed an activity of 7.18 and 6.25 high (Figure 7C). Finally, the one-cell stage C57BL/6 embryos were injected by the mixture of 25 ng/μl of sgRNA and 50 ng/μl of Cas9 mRNA, and then the gene-edited embryos were transferred into two pseudo pregnant foster mothers' endometrium.

Due to the random mutation feature of this technology, there were several heterozygous MPC1 mutation mice in F0. Five homozygous mutation mice fostered by the NO.17 mouse were obtained with the heterologous F0 mice mating experiments.

### Genomic DNA extraction, PCR, restriction digests assay and sequencing

Two weeks old mice were tagged by toe cut, and the MPC mutation identification in mice was performed with tail snip DNA extracted (OMEGA, D3396). The DNA was then used for PCR amplification with PCR reaction mixes containing 10μl Taq MasterMix (CWBIO, CW0682), 1μl of each primer (MPC1-DNA-Forward: AGCGGTCGTAAGGCTTCTCC, Reverse: TCCCCTGAGTCCTGCTGTCC), 2μl of DNA template and RNase-free water in a final volume of 20μl. The PCR Cycling conditions included an initial hot start at 94°C for 30 seconds, 38 cycles of 94°C /25 seconds, 60°C /20seconds and 72°C /25seconds, plus a final 72°C extension for 10 seconds.

The annealed PCR amplified DNA was treated with 0.5μl of T7E1 (New England BioLabs, M0302S) for 20 min at 37°C and then subjected to agarose gel electrophoresis. If the DNA sequences displayed mutations, heteroduplexes would be formed. The PCR products were sequenced by Sangon Biotech company (Shanghai, China). The gene-sequencing atlas was analysed by the Chromas software.

### Off-target sequencing experiment

For the off-target verification experiment, potential MPC1-g1 and MPC1-g2 off-targets were firstly identified according to Zhang Lab's webpage (http://crispr.mit.edu/) before PCR-based sequencing was performed.

The MPC1-g1 sgRNA target is at locus chr17:+8476773. There was a list of the off-targets for this sgRNA. We selected all the off-targets with hit score higher than 1 for further analyses, since those lower than 1 hit score off-target sites may have no mutations as reported [39]. There are two off-targets with hit score higher than 1 were needed to check in our study. These two were the locus at chr12:-112097058 and chr12:+78920516, with off-target hit scores of 100.0 and 49.2, respectively.

The MPC1-g2 sgRNA target sequence is at the same locus as shown in Figure 7. There were three potential off-targets with hit score higher than 1 for this sgRNA. These off-targets were at chr12:+78920523, chr12:-112097051 and chrX:+140020779, with off-target hit scores of 100.0, 26.8 and 1.5, respectively.

For the loci at chr12:+78920516 and chr12: +78920523, the following primers were applied for PCR amplification: Forward: CTGCCAACTGATAA TCTCTGGA; Reverse: GGCTTTACTGCCAACAGCAT. For the loci at chr12:-112097058 and chr12:-112097051, the following primers were applied for PCR amplification: Forward: GCTGTAAACACCCAAGCAGT; Reverse: AGAATAGCAACAGAGGGCGA. For the locus at chrX:+140020779, the primers were used in PCR amplification: Forward: GAGGGGGAAAGAAA CTATGAGA; Reverse: TGCCCATAGACCTCTACCAA. The PCR products were sequenced by Sangon Biotech company (Shanghai, China).

### Protein expression by western-blot

Lyses the tissues with mixed buffer of RIPA buffer and 1% PMSF (Thermo Scientific, West Palm Beach, FL) by vortexing and put on ice for about 30 minutes, and then centrifuged at 12,000 rpm for five minutes at 4°C. 30μg proteins were loaded to 10% SDS-PAGE and then transferred to PVDF membrane. 5% fat-free milk was used to block the membrane for two hours at room temperature, followed by incubated overnight at 4°C with a rabbit anti-mouse BRP44L (1:500, Novus) and BRP44 antibody (1:500, abcam). Then the blot was incubated with relevant second antibody for two hours (1:3000, CWBIO, Beijing, China) and detected by the enhanced chemiluminescence detection kit (ECL, Amersham, UK). The band density of the target protein was normalized by β-actin and calculated by the Bio-Rad Quantity One 1-D Analysis Software (Bio-Rad, Hercules, CA).

### Quantitative RT-PCR assay

When reached to 24 weeks old, the mice were executed and various tissues were lysed in TRIZOL (TAKARA, #9109) for RNAs extracted. Revert Aid First Strand cDNA Synthesis Kit (TAKARA, #RR047A) was used for cDNAs synthesis. The forward and reverse primers sequences are shown in Table 3. SYBR green master mix (ABI, #RR820A) was applied in the PCR amplifications. The cycling conditions were 95°C for 10 minutes; 40 cycles of 95°C for 15 seconds and 60°C for 60 seconds. The data were analyzed by The Step One Plus™ System (ABI).

**Table 3:** The qRT-PCR primer sequence

β-actin-F	5′-GTGCTATGTTGCTCTAGACTTGG-3′
R	5′-ATGCCACAGGATTCCATACC-3′
MPC1-F	5′-GGTACAACCTCGAAACTGG-3′
R	5′-TCAAGAGCTGGTCCTTGTACC-3′
MPC2-F	5′-GGGACCTTTTCCTCACGTCC-3′
R	5′-GTTCTGGGACCTGCTGGATG-3′
Fbp-F	5′-TCAACTGCTTCATGCTGGAC-3′
R	5′-AGTCCTTGGCATAACCCTCA-3′
Ppargc 1a-F	5′-CCGCAATTCTCCCTTGTATG-3′
R	5′-CTCTTGAGCCTTTCGTGCTC-3′
G6pc-F	5′-GTTTCGCGCTTGGATTCTAC-3′
R	5′-GTCAAGGTGGACCCATTCTG-3′
Fasn-F	5′-GCCCTCATCGACCTACTGAC-3′
R	5′-GGAGAGACAGCCATCTGCAT-3′
Acc-F	5′-CCGAGCAAGGGATAAGTTTG-3′
R	5′-ATGGGATGGCAGTAAGGTCA-3′
Pparg-F	5′-AGACCACTCGCATTCCTTTG-3′
R	5′-CATTGGGTCAGCTCTTGTGA-3′
Srebf1-F	5′-ACCACCGTCACTTCCAGCTA-3′
R	5′-TGGTCCTGATTGCTTGTCAG-3′

### IHC detection

IHC detection of mouse liver MPC1 and MPC2 was performed with the Dako Envision FLEX+ system (K8012; Dako, Glostrup, Denmark). Paraffin slices were de-paraffinized and dehydrated in xylene and ethanol series, respectively. Epitope unmasking was performed with microwaving antigen retrieval in citrate buffer (pH 6.0) for 15 minutes before being returned to room temperature and washed in PBS. Unspecific blocking was operated by peroxidase blocking (Dako) for five minutes. The sections with primary antibody (BRP44L, 1:700; BRP44, 1:500) were incubated at 4°C for 14h. The second antibody and HRP were used for 15 minutes and 30 minutes in sequence. Diaminobenzidine tetrahydrochloride (DAB) was used to stain the slides for 10 minutes and counterstained with hematoxylin, dehydrated and mounted.

### Bodyweight and metabolic profile

All mice at an age of two weeks were toe-tagged, and all the MPC1 mutation mice were sex- and age-matched with the same old wild-type mice. MPC1 KO and WT mice were fed with a standard diet (SD, 5.5% fat content) and weighed from two weeks of age to 14 weeks of age. On week 15, intraperitoneal glucose tolerance test (IPGTT) was performed for the mice. The blood glucose was monitored at 0, 15, 30, 60 and 120 minutes using a Bayer glucometer. On week 16, an IPITT was performed for all the mice, and the blood glucose was measured at 0, 15, 30, 60 and 120 minutes [24].

### Statistical analyses

Repeated measures (at least three times) were analyzed by one-way ANOVA and Student's T test using PrismDemo software. All experiments data were shown as mean ± SEM. It was considered to be statistically significant for p<0.05.
